# Management of the Insane

**Published:** 1862-03

**Authors:** George C. McFarland

**Affiliations:** A Student of Rush Medical College


### MANAGEMENT OF THE INSANE.

By GEORGE C. McFARLAND,
*A Student of Rush Medical College.*

The treatment of insanity of late years has become more of a speciality. Thus the student of Medicine when he reads his works on practice merely glances at the subject, and the practitioner himself recommends his patients to the various asylums constructed for their benefit, and little is known in regard to their treatment and management by the profession. Now we would throw overboard a large list of drugs contained in the materia medica. Medicines are of no use whatever. To successfully treat the insane, the asylums are of course the proper place. The patient should not have the idea that he is going to a prison, and be treated as a felon, but let him know the facts; treat him in person, as if he were sane; let the physician in attendance not assume a manner foreign to his disposition; let him approach his patient in a quiet, self-possessed manner. Bring the patient in contact with sane men; let books and periodicals be thrown in his way, and soon he will rouse and the case seem hopeful. We would cite a case that came under our observation.

T. K., brought to the asylum some years ago, having committed a homicide. At the time of his admittance he was very morose and sought to kill all his fellow men; seldom spoke, and when he did would call for a butcher knife to cut his way out. Books were handed him, and in his solitude he acquired a taste for reading; soon after he was taken out to do light work; soon a change was manifest, and at the present time he fills his accustomed seat at the divine service and takes much interest in the proceedings. So much improvement is seen we consider him in a fair way of recovery.

Now we think this treatment could be well introduced in poor houses, for it is well known that, for want of room, our State institution does not come up to the requirements of the State, and poor houses of the various counties become their retreat, and the arrangements they have for the care of the insane is wholly inefficient. Thus we have seen men chained to the floor, a total want of food and clothing, rendering them objects of commiseration.

A man was admitted to the hospital, having had previously for his habitation a hole in the ground, roofed over, with an opening simply large enough for food to be passed down. For years he remained in this condition, and became an object of dread to the people. At last the real estate owners thought this man injured the sale of their lands by his hideous cries, and pronounced him a nuisance. A public meeting was called. Some were for killing him; others again were for constructing a similar den in the woods. The Legislature, however, being in session, some good man caused to be appropriated a sum of money, and he was placed in an Insane Hospital. Coming in contact with men, he gradually began to improve, and at the present time, he is gaining his livelihood in the town as a laborer. Finally, we would say in the management of the insane, let all drugs be discarded; let the patient feel that he is a man; wait upon him in words of kindness, not in violence and deceitfulness; rule him by love, not fear, and we think those called to treat the insane will meet with success. Occupation which requires thought is far preferable, in a curative point of view, to that which requires none. Thus the artisan employment for men and household employments for women, are much more useful as curative agencies, than the use of the spade and hoe for the former, or the needle for the latter.

Mechanical restraint in the management of the insane is fast going out of date. It was not long since that shower baths filled every ward and the chief employment of the more convalescent patients was in constructing the straight-jacket for controlling the more violent. Now we would discard these wholly. However, where the patient is suicidal, and seeks to injure himself and others, then perhaps a comfortable straight-jacket may be admissable. In conclusion, we would say, this subject of moral treatment that we have suggested may be worthy of larger consideration in the management of the insane.

[*Remarks.*—The writer of the foregoing will be recognized by many of our readers as a son of the sagacious and popular Superintendent of the Illinois State Insane Asylum. While there is no especial novelty in the views advanced, the extreme cases briefly narrated will serve well to illustrate the benign influences of the modern treatment of insanity, in strong contrast with that in olden time. There is an amount of suffering and horror among the pauper insane, even of this State, which would startle the most stolid were it fully depicted. One assertion of our correspondent is too sweeping. "Drugs" are not to be entirely discarded in the treatment of the insane. The advances of the present time in physiology, pathology and treatment have shown that the material lesions, centric and ex-centric, which originate or intensify mental disorders, may be reached, to a limited extent, by skilful medication. Insanity is always a result of material lesion. It is not a merely spiritual affair; but, as in other maladies, hygiene and correct regimen are leading methods of cure.—A.]

